# Porphyrin-grafted Lipid Microbubbles for the Enhanced Efficacy of Photodynamic Therapy in Prostate Cancer through Ultrasound-controlled *In Situ* Accumulation: Erratum

**DOI:** 10.7150/thno.100601

**Published:** 2024-07-23

**Authors:** Yujia You, Xiaolong Liang, Tinghui Yin, Min Chen, Chen Qiu, Chuang Gao, Xiaoyou Wang, Yongjiang Mao, Enze Qu, Zhifei Dai, Rongqin Zheng

**Affiliations:** 1 Department of Medical Ultrasonic, The Third Affiliated Hospital of Sun Yat-sen University, Guangzhou 510630, China; 2 Department of Ultrasound, Peking University Third Hospital, Beijing 100191, China; 3 Department of Biomedical Engineering, College of Engineering, Peking University, Beijing 100871, China

The authors regret to find an error in the published version of Figure 4A, where the lower right image was misused. After carefully re-verifying the original data, the authors identified a labeling error that occurred during the image capture process. It turns out that the lower left and right images actually belong to the same experimental group but were mistakenly categorized into different groups (LFUS+ and LFUS-) due to labeling errors. The corrected version of Figure 4A appears below. The corrections made in this erratum do not affect the original conclusions. The authors apologize for any inconvenience that the errors may have caused.

## Figures and Tables

**Figure 4A:**
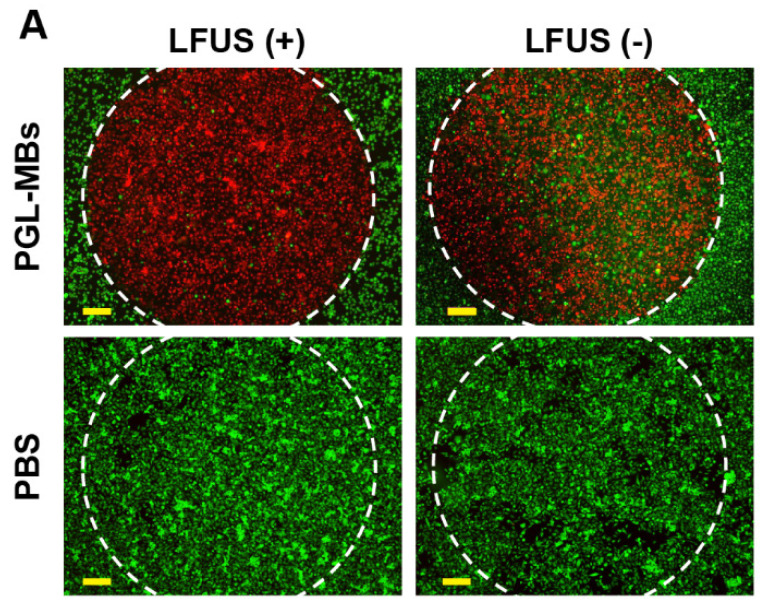
Correct image.

